# Supplementary material for: Phosphoproteomics data-driven signalling network inference: Does it work?
Source: Comput Struct Biotechnol J. 2022 Dec 15;21:432–43. doi: 10.1016/j.csbj.2022.12.010 (PMC9798138; doi:10.1016/j.csbj.2022.12.010)
Supplement: Supplementary data 1 — Fig. S1. Time lagged associations from the synthetic data. Fig. S2. Distribution plots of Fisher’s exact estimated odds ratio for enrichment of true positive hits in top 20% of predicted associations. Fig. S3. Distribution plots of log10 AUPRC/Baseline ratio for interactome derived association. Fig. S4. Distribution plots of AUC metrics. Fig. S5. Distribution plots of true positive kinase substrate rank in inferred associations and randomised kinase substrate associations. Fig. S6. Distribution plots of log10 estimate odds ratio for interactome derived substrate-substrate associations. Fig. S7. Distribution plots of log10 AUPRC/baseline ratio for interactome derived substrate-substrate associations. Fig. S8. Distribution of sequence similarity scores between pairs of substrates regulated by the same kinase. Fig. S9. Heatmap of AUC performance metrics across all datasets and association measures used for evaluation. Fig. S10. Heatmap of Precision recall to baseline metric ratio. Fig. S11. Heatmap of estimate odds ratio from the Fisher’s exact top 20% TP enrichment test. Supplementary Table S1. Datasets used in this study and the data processing steps applied to each dataset. Supplementary Table S2. The median values of 100k sampled TP and TN sequence similarity between substrate-substrate phosphosites [file mmc1.pdf]

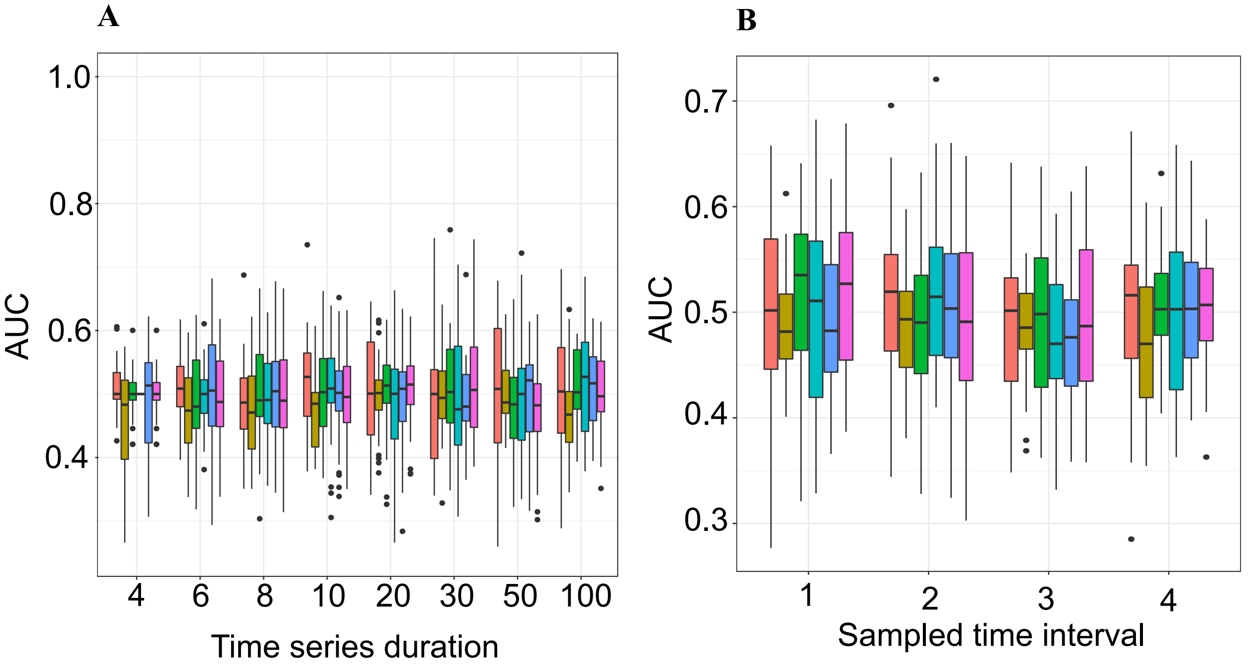

Figure S1. Time lagged associations from the synthetic data. A. AUC distribution when data was sampled using varying time duration as displayed in the x axis **B.** AUC distributions when we performed intermittent sampling every 1,2,3 or 4 timepoints from data of length 100 timepoints

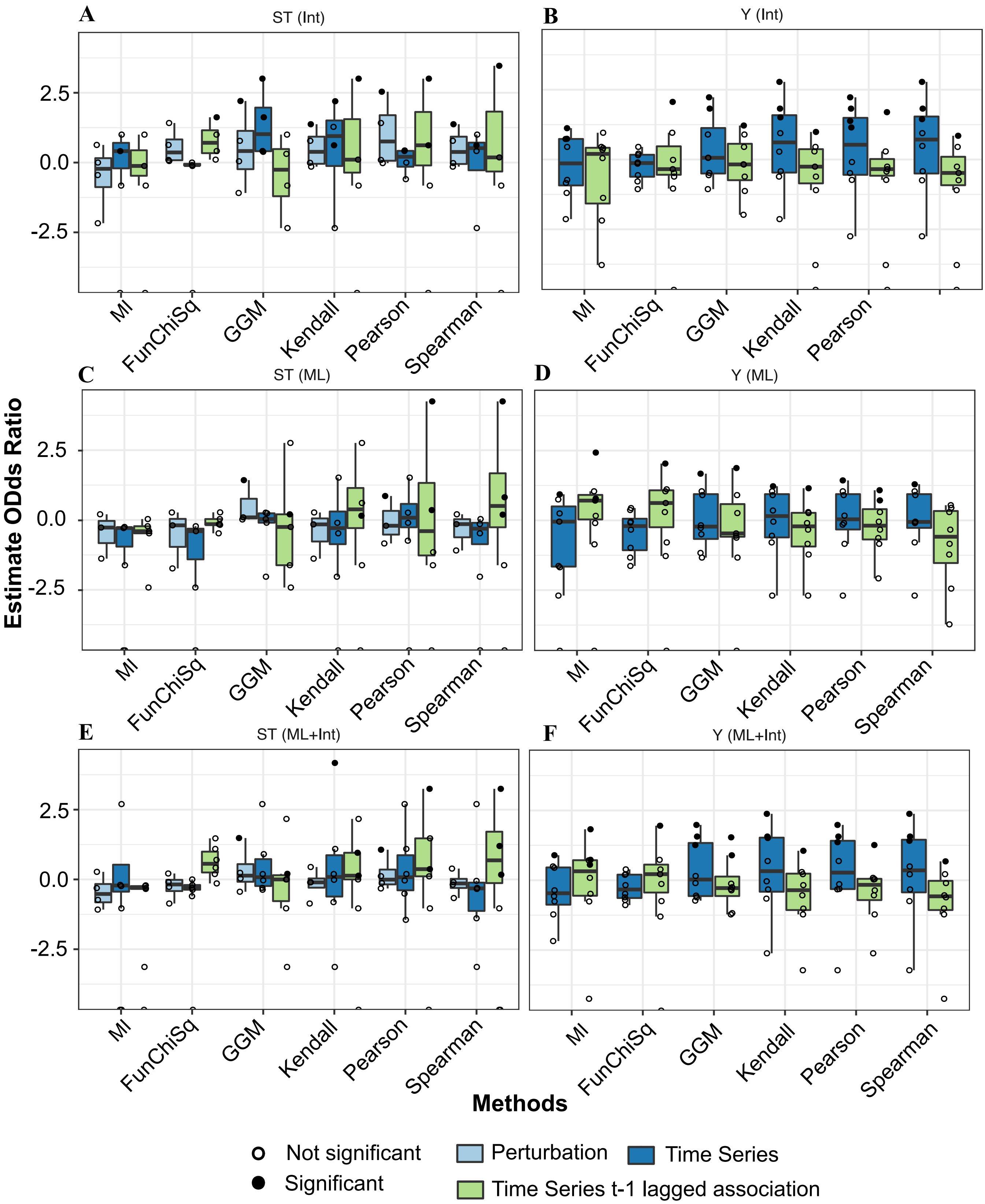

Figure S2. **Distribution plots of log10 AUPRC/Baseline ratio for interactome derived association**. **A.** S/T kinase substrate associations **B.** Y kinase substrate associations **C.** Machine learning derived S/T kinase substrate associations **D.** Machine learning derived Y kinase substrate associations. **E.** Combined interactome and machine learning derived S/T kinase substrate associations **F.** Combined interactome and machine learning derived Y kinase substrate associations. The black filled in points on the boxplots indicates statistical significance from the permutation analysis with p-value < 0.05.

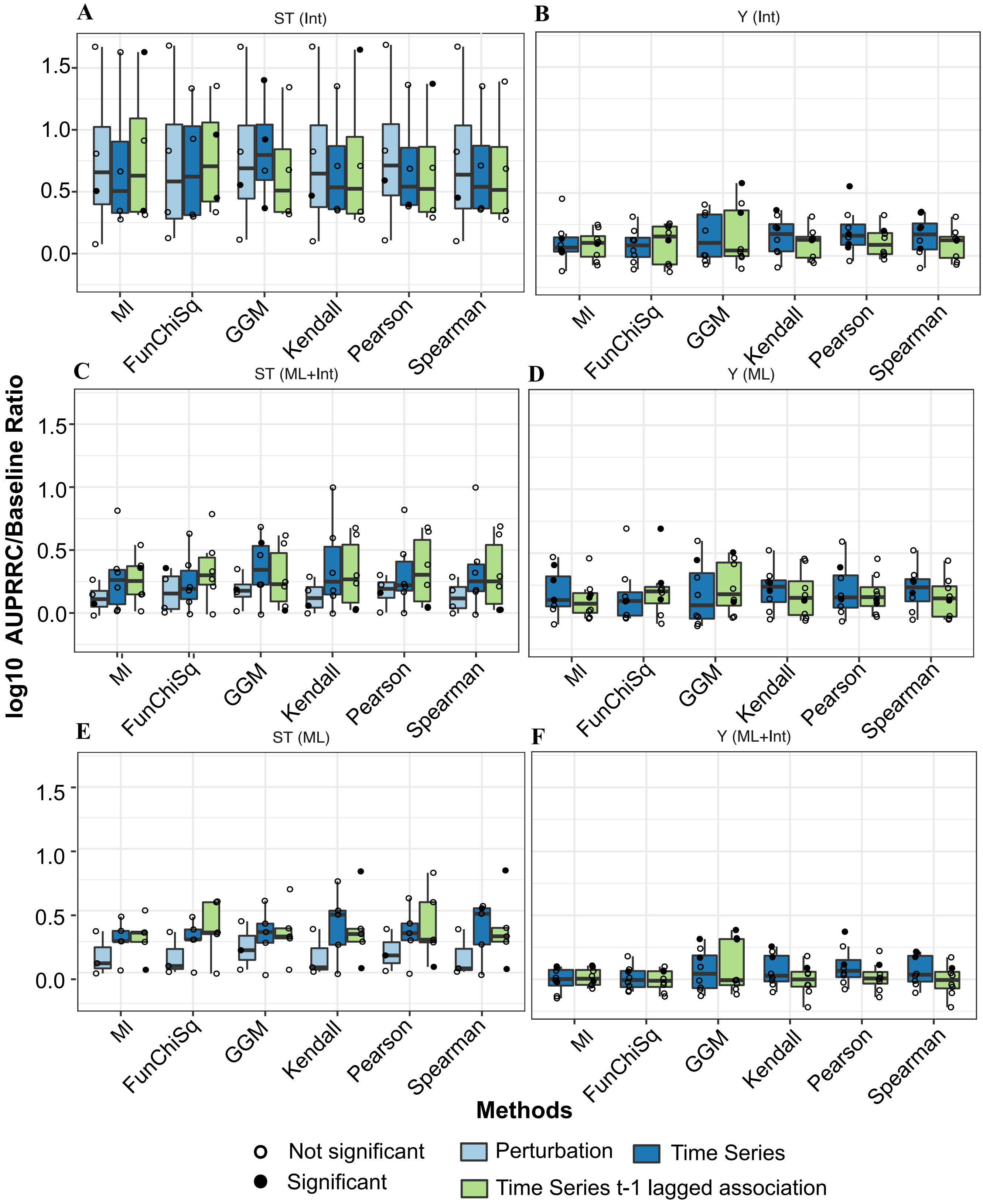

Figure S3. Distribution plots of Fisher's exact estimated odds ratio for enrichment of true positive hits in top 20% of predicted associations for A. S/T kinase substrate associations **B.** Y kinase substrate associations **C.** Machine learning derived S/T kinase substrate associations **D.** Machine learning derived Y kinase substrate associations **E.** Combined interactome and machine learning derived S/T kinase substrate associations **F.** Combined interactome and machine learning derivedY kinase substrate associations. The black filled in points on the boxplots indicates statistical significance from the permutation analysis with p-value < 0.05.

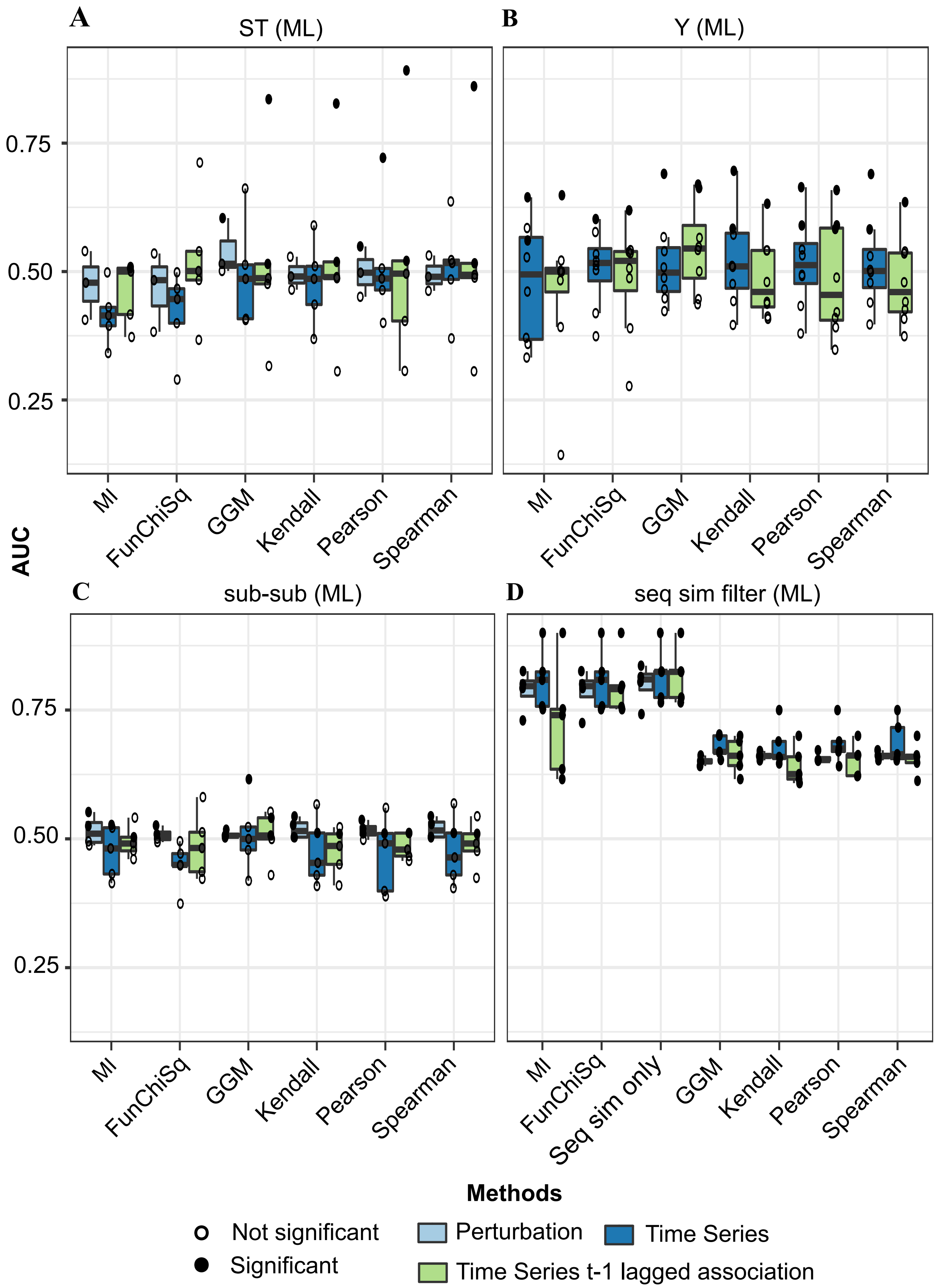

Figure S4: **Distribution plots of AUC metrics for A.** S/T kinase substrate associations **B.** Y kinase substrate associations **C.** Pairwise substrate-substrate associations regulated by the same kinase **D.** Pairwise substrate substrate associations regulated by the same kinase with sequence similarity filter. The black filled in points on the boxplots indicates statistical significance from the permutation analysis with p-value < 0.05.

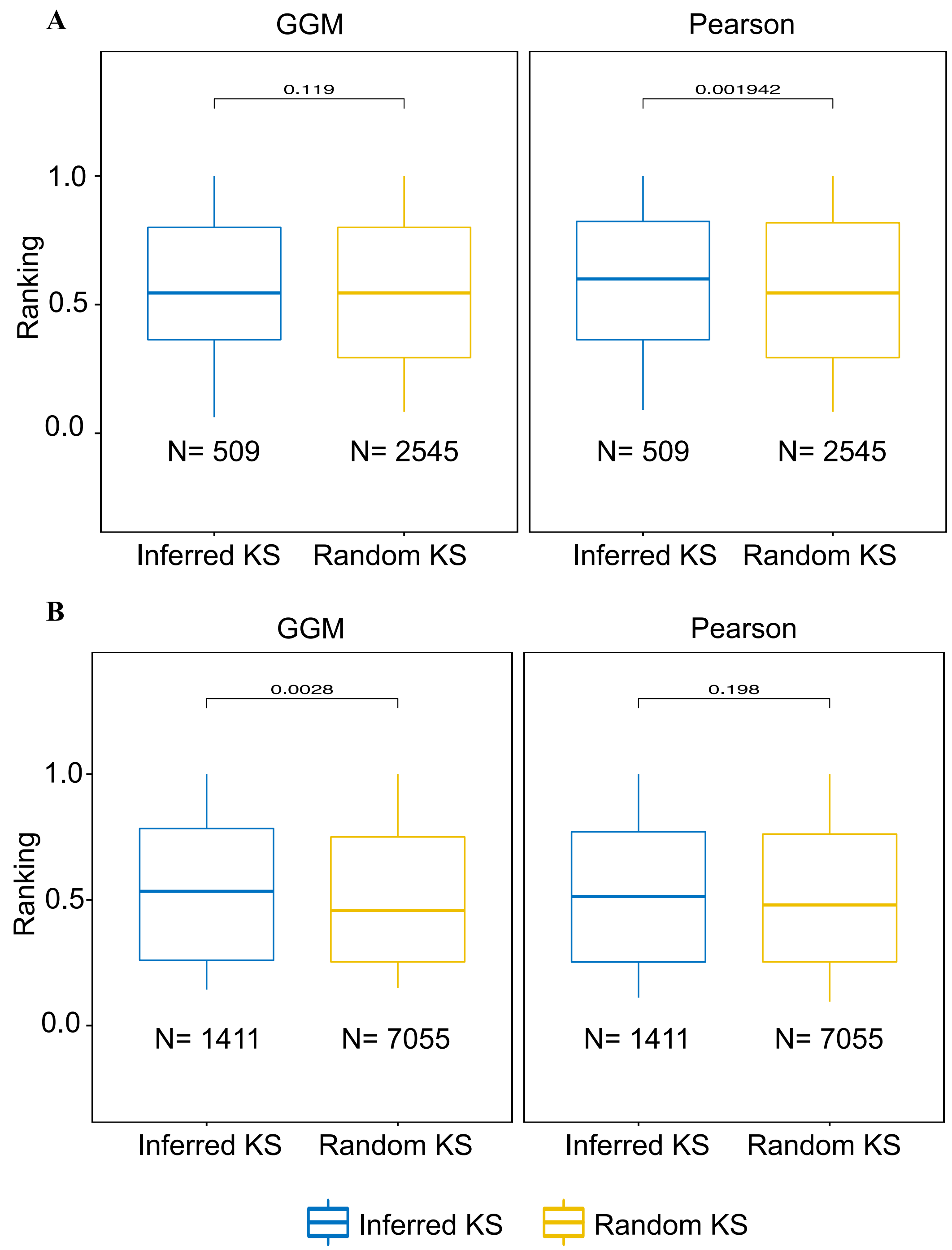

Figure S5: Distribution plots of true positive kinase substrate rank in inferred associations and randomised kinase substrate associations A. GGM and Pearson distribution of Y kinase substrate associations **B.** GGM and Pearson distribution of S/T kinase substrate associations. A wilcoxon rank-sum test was performed for statistical significance.

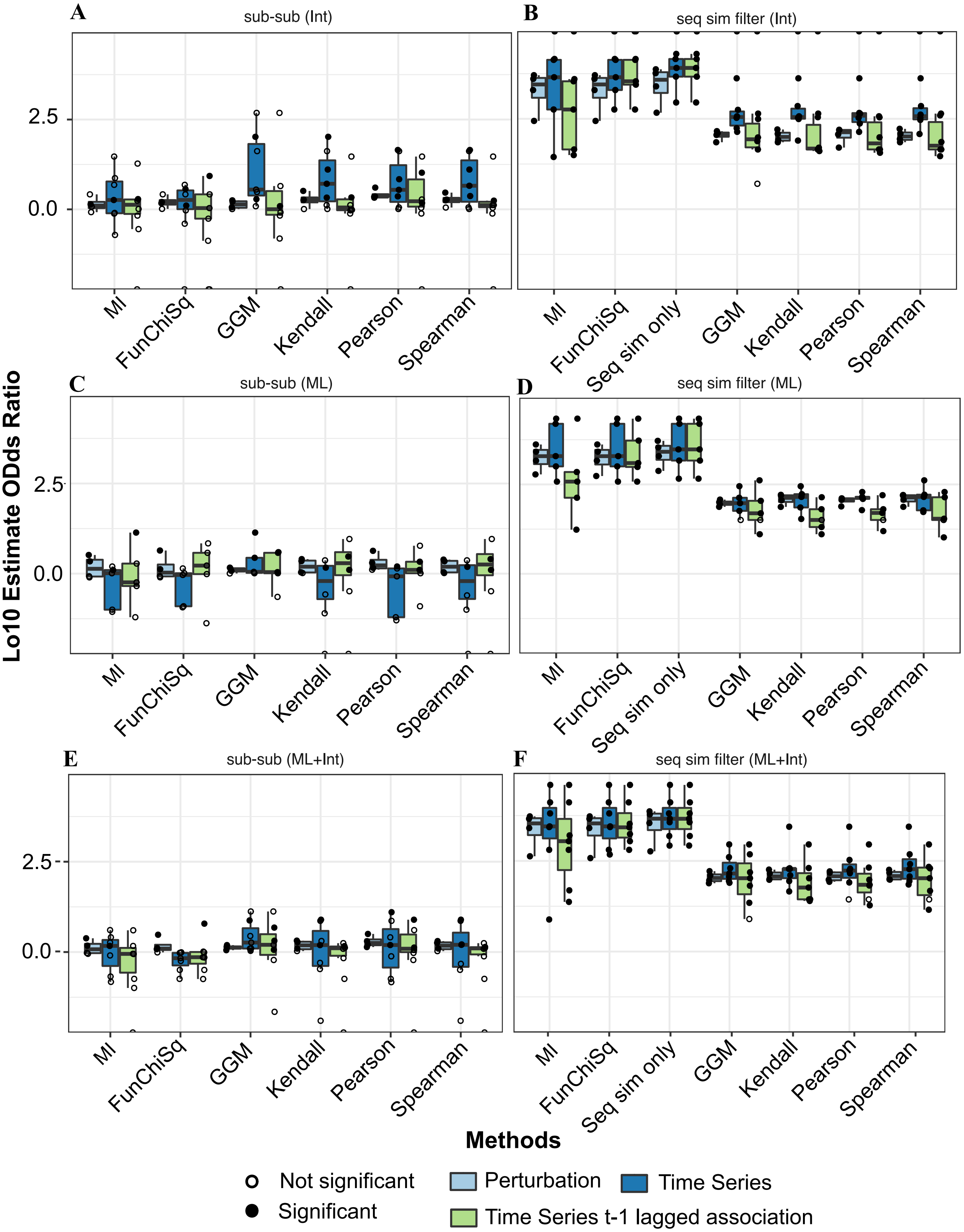

Figure S6: Distribution plots of log10 estimate odds ratio for interactome derived
A. Substrate-substrate associations **B.** Substrate-substrate associations with sequence similarity filter **C.** Machine learning derived substrate-substrate associations **D.** Machine learning derived substrate-substrate associations with sequence similarity filter **E.** Combined interactome and machine learning derived substrate-substrate associations **F.** Combined interactome and machine learning derived substrate-substrate associations with sequence similarity filter. The black filled in points on the boxplots indicates statistical significance from the permutation analysis with p-value < 0.05.

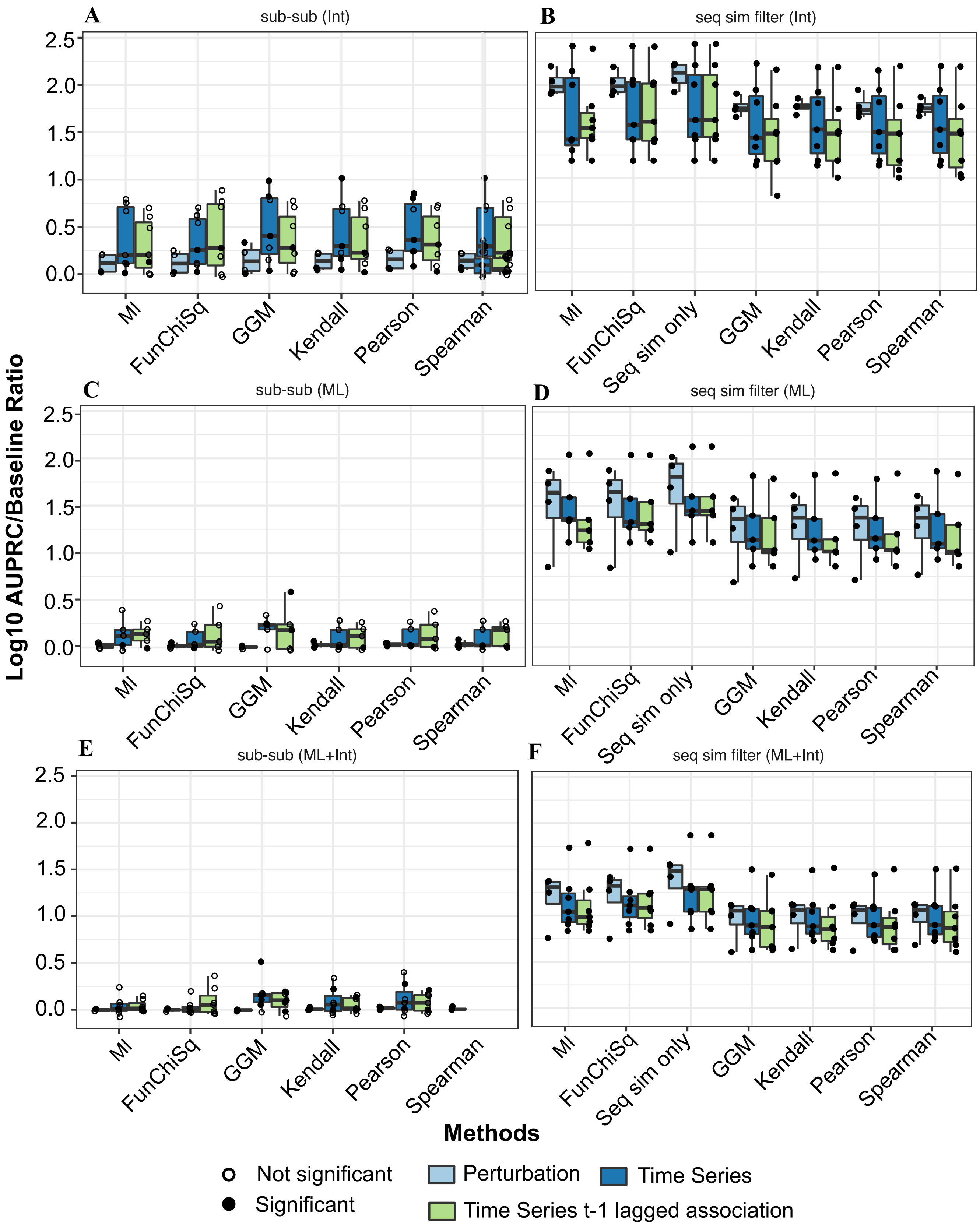

Figure S7: Distribution plots of log10 AUPRC/baseline ratio for interactome derived A. Substrate-substrate associations **B.** Substrate-substrate associations with sequence similarity filter **C.** Machine learning derived substrate-substrate associations **D.** Machine learning derived substrate-substrate associations with sequence similarity filter **E.** Combined interactome and machine learning derived substrate-substrate associations **F.** Combined interactome and machine learning derived substrate-substrate associations with sequence similarity filter. The black filled in points on the boxplots indicates statistical significance from the permutation analysis with p-value < 0.05.

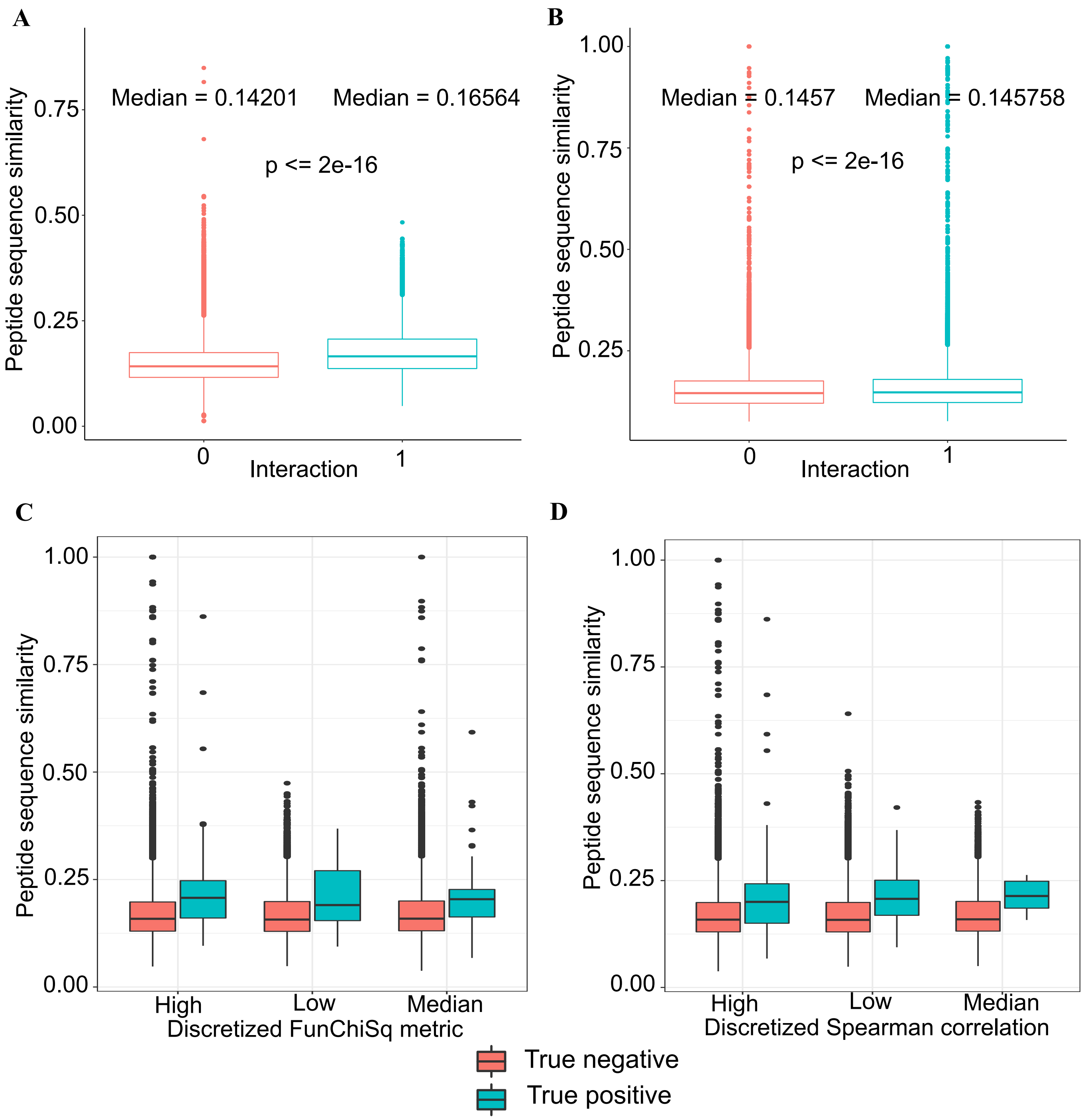

Figure S8: Distribution of sequence similarity scores between pairs of substrates regulated by the same kinase. An interaction value of 1 represents a TP, and an interaction value of 0 represents a TN. The pair of distributions plots represent **A.** Serine/threonine phosphosites **B.** Tyrosine phosphosites. Discretized association measures across three levels (High, Medium, Low) with respect to sequence similarity for true positive and true negative interactions from the phosphositeplus database. Plots represent **C.** Discretized FunChiSq **D.** Pearson correlation measures.

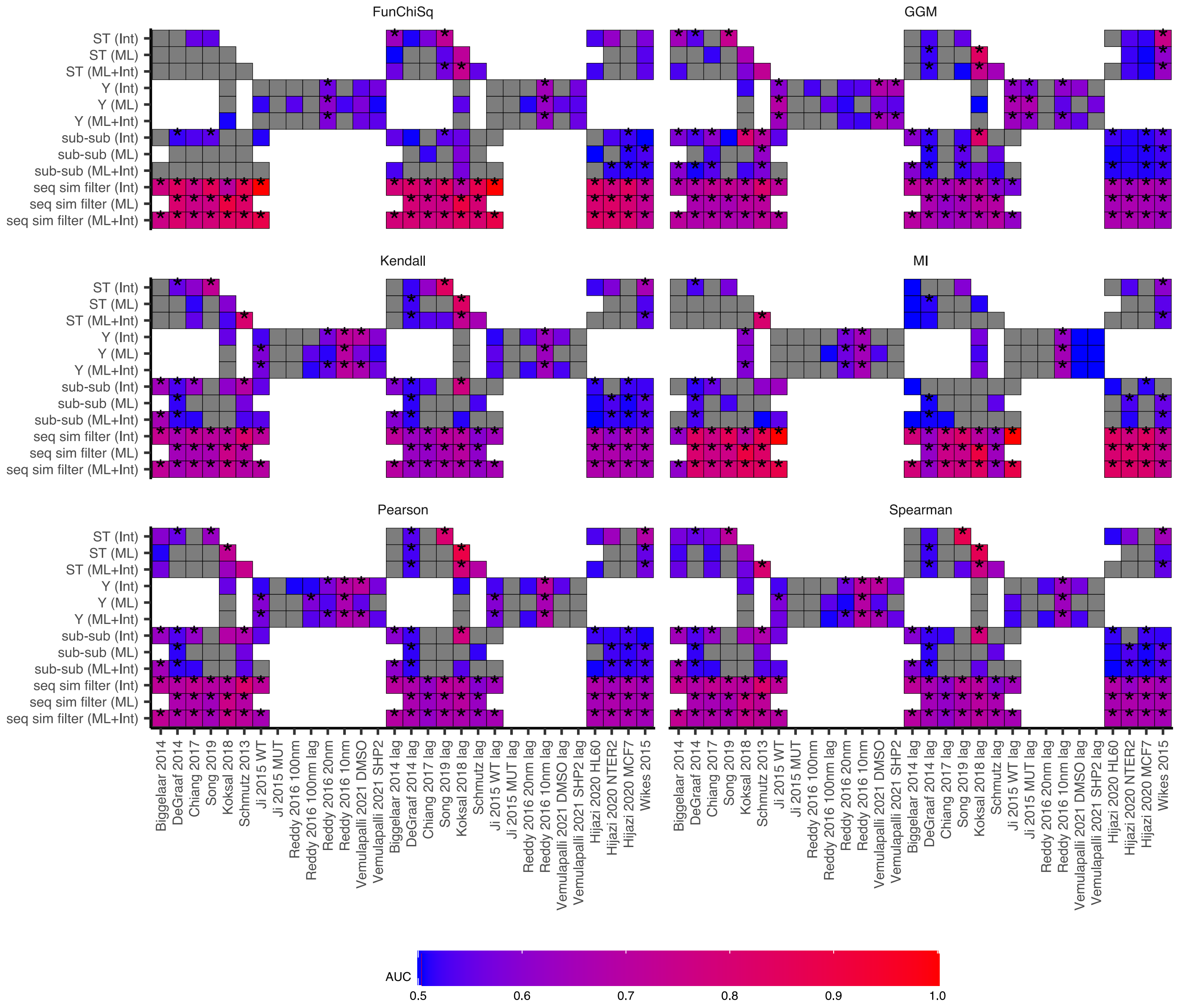

Figure S9: Heatmap of AUC performance metrics across all datasets and association measures used for evaluation. An asterix indicates significance from the permutation analysis with p-value < 0.05. Colourscale of grey indicates datasets with AUC < 0.5.

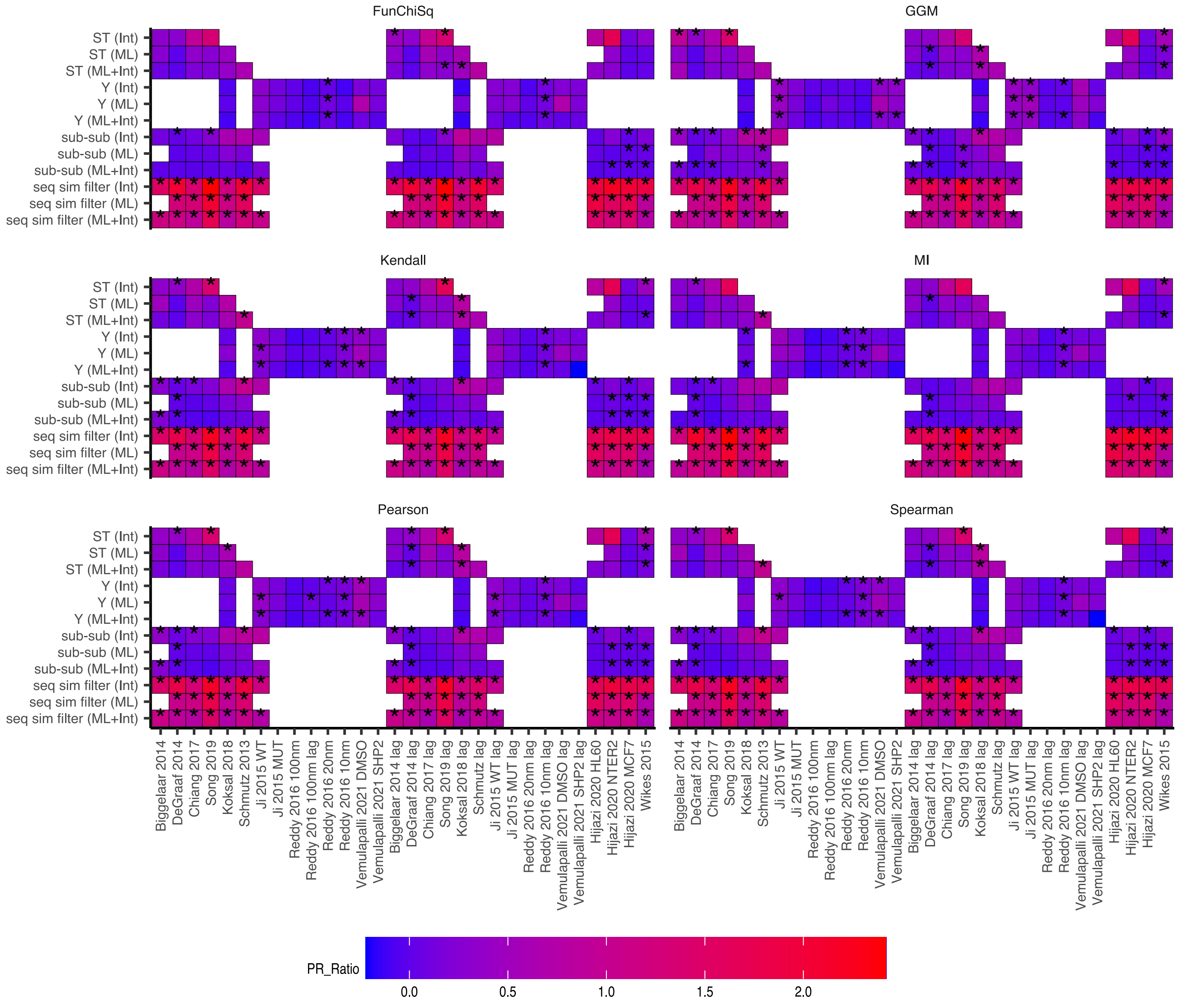

Figure S10: Heatmap of Precision recall to baseline metric ratio. The ratio used in the plot has been log10 scaled. An asterix indicates statistical significance from the permutation analysis **with p-value < 0.05.**

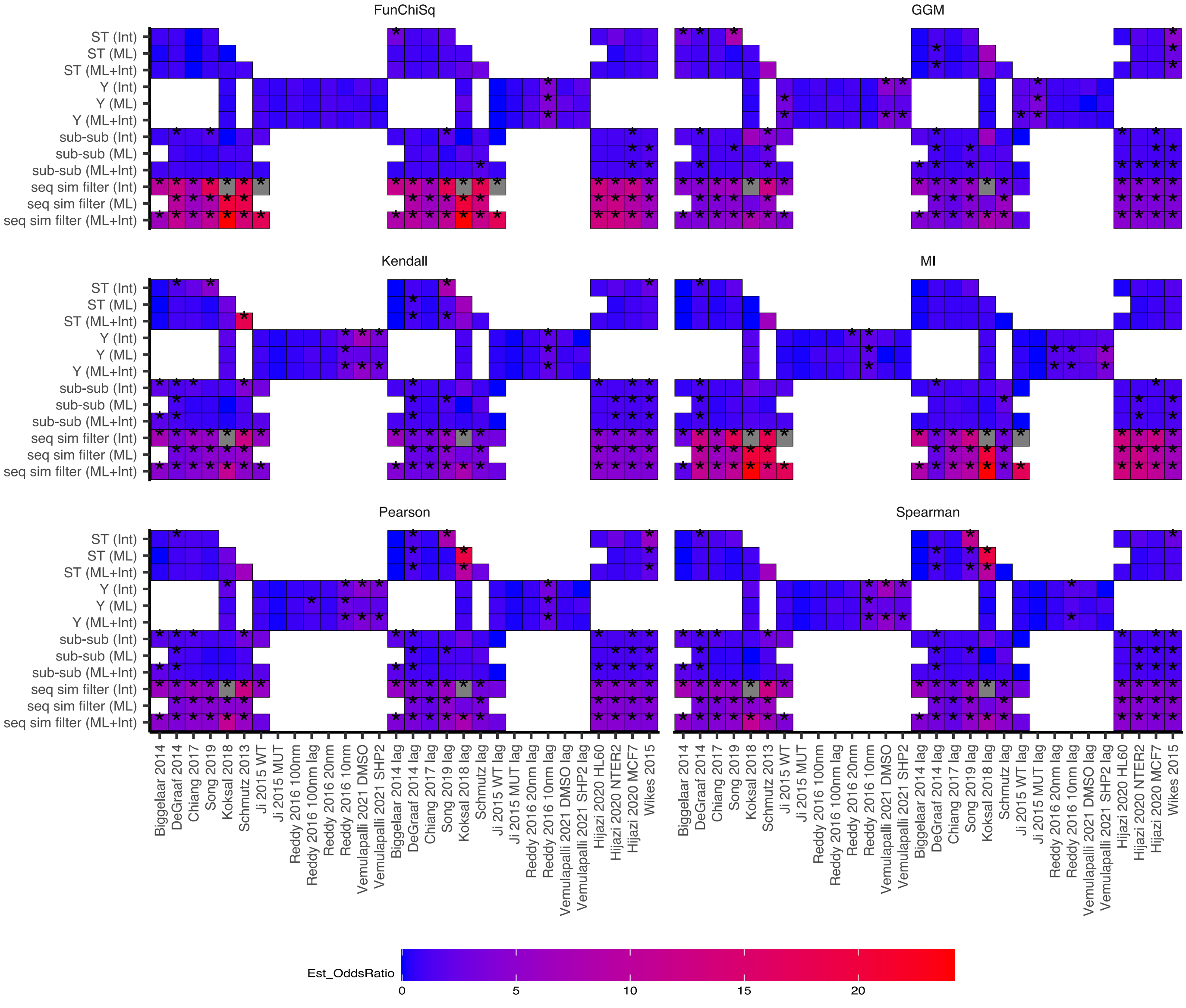

Figure S11: Heatmap of estimate odds ratio from the Fisher's exact top 20% TP enrichment test. An asterix indicates statistical significance from the permutation analysis with p-value < 0.05.

Supplementary Table S1: Datasets used in this study and the data processing steps applied to each dataset

Dataset	Reference	Processing
Koksal et al 2018	[1]	This dataset was provided as a concatenated dataset across three biological replicates. There are three technical replicates for each biological replicates (3 biological replicates in total). Across the three biological replicates, 1068 peptides were present in all three replicates. This dataset was provided pre-processed (median normalized) as three biological replicates. Each row for each replicate was normalized to the row mean to minimise batch effect variations between the biological replicates.
Vemulapalli et al 2021	[2]	This dataset has 2 replicates, under two conditions: DMSO stimulated, and SHP2 stimulated. These datasets are column median normalised and row mean normalised across each TMT 11plex replicate set. The phosphosites across both replicates were matched.
Ji et al 2015	[3]	Silac labelled cells. Two jurkat cell lines used: mutant (J14–2D1) and WT (J14–76-11). Identical phosphopeptides from the same protein were summed and median normalized again. The data was row mean normalized per replicate set. Replicate 5 was omitted from the analysis as one time point was missing.
Song et al 2019	[4]	This is TMT dataset of a neural progenitor cell line ReN cell line. There are two replicates of the proteome and the phosphoproteoeme. Both datasets were median column nornalised and row mean normalised. A phosphoproteome to proteome ratio for each time point was generated.
Biggelaar et al 2014	[5]	This is a SILAC dataset of thrombin signalling in human endothelial cells. No normalisation or correction is performed on this dataset as each time point sample was provided as a ratio to t=0 stimulation.

De Graaf et al 2014	[6]	This is a label-free dataset of Jurkat T cells stimulated by prostglandin and measured over six time points. The normalised average signal intensity data was used and a fold change filter was applied to select the differentially expressed phosphosites. Data was normalised to t=0 and analysed as ratios.
Reddy et al 2016	[7]	TMT labelled MCF-10A cells stimulated with different concentrations of EGF (0.2, 0.4, 1, 2.5, 5, 10, 20, 100 nM EGF) measured over 80 seconds. Each dataset had 9 timepoints, but no replicates. The data provided in the supplementary materials was median normalized and normalized to a control channel to generate ratios for each phosphosite.
Wilkes et al 2015	[8]	Label-free datasets. This dataset comes preprocessed with log2 fc ratios against a control sample and p-values. Data was filtered based on the following conditions: 20% or more of the samples in each row needs to have p-value <c0.05 and 3 or more sites are required to have an absolute log2fc ratio greater than 2.
Hijazi et al 2020	[9]	Label-free datasets. This dataset comes preprocessed with log2 fc ratios and p-values. Data filtering steps used for Wilkes et al 2015 was also used for this dataset.
Schmutz et al 2013	[10]	This is a label-free dataset with three replicates in which the phosphoproteome (log2 ratios to t=0) was normalized to the proteome (log2 ratios to t=0) across 4 timepoints.
Chiang et al 2017	[11]	Dataset came as log2 (phos/prot) ratios with three replicates. No processing was required for this dataset.

Supplementary Table S2: The median values of 100k sampled TP and TN sequence similarity between substrate-substrate phosphosites. The TP substrate-substrate associations were excluded for each dataset and then sampled randomly to estimate the median values of both the TP and TN distributions. The Wilcoxon-rank sum test was performed to test for significance between both distributions.

Dataset	TP.Median	TN.Median	Wilcoxon p-value	Reference
Biggelaar 2014	0.173395	0.14431272	0	[5]
DeGraaf 2014	0.17218921	0.14450262	0	[6]
Hijazi 2020 HL60	0.17118605	0.14404027	0	[9]
Hijazi 2020 MCF7	0.17037554	0.14474937	0	[9]
Hijazi 2020 NTER2	0.16993827	0.14460456	0	[9]
Koksal 2018	0.16996732	0.1446798	0	[1]
Ji 2015 WT	0.17002546	0.14404027	0	[3]
Song 2019	0.17002546	0.14473684	0	[4]
Wilkes 2015	0.16993827	0.14473346	0	[8]
Schmutz 2013	0.16993827	0.14474937	0	[10]
Chiang 2017	0.16996732	0.14474937	0	[11]

References:

[1] Köksal AS, Beck K, Cronin DR, McKenna A, Camp ND, Srivastava S, et al. Synthesizing Signaling Pathways from Temporal Phosphoproteomic Data. Cell Reports 2018;24:3607–18. https://doi.org/10.1016/j.celrep.2018.08.085.

[2] Vemulapalli V, Chylek LA, Erickson A, Pfeiffer A, Gabriel K-H, LaRochelle J, et al. Time-resolved phosphoproteomics reveals scaffolding and catalysis-responsive patterns of SHP2-dependent signaling. ELife 2021;10:e64251. https://doi.org/10.7554/eLife.64251.

[3] Ji Q, Ding Y, Salomon AR. SRC Homology 2 Domain-containing Leukocyte Phosphoprotein of 76 kDa (SLP-76) N-terminal Tyrosine Residues Regulate a Dynamic Signaling Equilibrium Involving Feedback of Proximal T-cell Receptor (TCR) Signaling. Mol Cell Proteomics 2015;14:30–40. https://doi.org/10.1074/mcp.M114.037861.

[4] Song Y, Subramanian K, Berberich MJ, Rodriguez S, Latorre IJ, Luria CM, et al. A dynamic view of the proteomic landscape during differentiation of ReNcell VM cells, an immortalized human neural progenitor line. Sci Data 2019;6:190016. https://doi.org/10.1038/sdata.2019.16.

[5] van den Biggelaar M, Hernández-Fernaud JR, van den Eshof BL, Neilson LJ, Meijer AB, Mertens K, et al. Quantitative phosphoproteomics unveils temporal dynamics of thrombin signaling in human endothelial cells. Blood 2014;123:e22–36. https://doi.org/10.1182/blood-2013-12-546036.

[6] de Graaf EL, Kaplon J, Mohammed S, Vereijken LAM, Duarte DP, Redondo Gallego L, et al. Signal Transduction Reaction Monitoring Deciphers Site-Specific PI3K-mTOR/MAPK Pathway Dynamics in Oncogene-Induced Senescence. J Proteome Res 2015;14:2906–14. https://doi.org/10.1021/acs.jproteome.5b00236.

[7] Reddy RJ, Gajadhar AS, Swenson EJ, Rothenberg DA, Curran TG, White FM. Early signaling dynamics of the epidermal growth factor receptor. Proc Natl Acad Sci U S A 2016;113:3114–9. https://doi.org/10.1073/pnas.1521288113.

[8] Wilkes EH, Terfve C, Gribben JG, Saez-Rodriguez J, Cutillas PR. Empirical inference of circuitry and plasticity in a kinase signaling network. PNAS 2015;112:7719–24. https://doi.org/10.1073/pnas.1423344112.

[9] Hijazi M, Smith R, Rajeeve V, Bessant C, Cutillas PR. Reconstructing kinase network topologies from phosphoproteomics data reveals cancer-associated rewiring. Nat Biotechnol 2020. https://doi.org/10.1038/s41587-019-0391-9.

[10] Schmutz C, Ahrné E, Kasper CA, Tschon T, Sorg I, Dreier RF, et al. Systems-Level Overview of Host Protein Phosphorylation During Shigella flexneri Infection Revealed by Phosphoproteomics *. Molecular & Cellular Proteomics 2013;12:2952–68. https://doi.org/10.1074/mcp.M113.029918.

[11] Chiang C-K, Tworak A, Kevany BM, Xu B, Mayne J, Ning Z, et al. Quantitative phosphoproteomics reveals involvement of multiple signaling pathways in early phagocytosis by the retinal pigmented epithelium. Journal of Biological Chemistry 2017;292:19826–39. https://doi.org/10.1074/jbc.M117.812677.
